# Regional Optimization Dynamic Algorithm for Node Placement in Wireless Sensor Networks

**DOI:** 10.3390/s20154216

**Published:** 2020-07-29

**Authors:** Yijie Zhang, Mandan Liu

**Affiliations:** Key Laboratory of Advanced Control and Optimization for Chemical Processes, Ministry of Education, East China University of Science and Technology, No. 130, Meilong Road, Shanghai 200237, China; y20140084@mail.ecust.edu.cn

**Keywords:** Wireless Sensor Networks, node placement, regional optimization

## Abstract

Node placement is one of the basic problems in a Wireless Sensor Network (WSN). During the operation of a WSN, sensor nodes may fail or die suddenly, which may lead to a coverage hole. To solve this problem, the node placement needs to be re-optimized. The dimensions of node placement optimization are high because of the large node number. In view of this defect, a regional optimization dynamic algorithm is put forward. In this paper, the regional optimization problem of node placement is modeled, and a regional optimization dynamic algorithm with a mixed strategy for node placement (MRDA) is proposed. Simulation experiments are carried out for the proposed algorithm and other comparison algorithms. Results of experiments show that the proposed algorithm can greatly reduce the dimensions and narrow the search range, with a significant improvement in the search performance and convergence speed.

## 1. Introduction

The Wireless Sensor Network (WSN) is a distributed sensor network that comprises several sensor nodes deployed in a specified area for data acquisition, processing and communication [1]. WSN has been one of the most popular research fields in recent years. It has been widely applied in agriculture, industry, health care, environment and so on [2,3,4].

Node placement can maximize the coverage of WSN while meeting other application requirements by deploying the position of sensor nodes. It can be classified as static node placement and dynamic node placement [5]. The static node placement is mainly used to determine the initial node placement to construct a WSN. The dynamic node placement can use mobile nodes to adjust the existing node placement whenever necessary.

Presently, the research on node placement mainly focuses on static node placement. However, in practical application, node fault or death may occur during the network operation, resulting in a coverage hole. The dead or fault nodes can be detected by analyzing the data of sink node [6]. The node placement needs to be re-optimized in this situation.

Coverage is one of the most important problems in WSN [7]. There are two main methods to repair the coverage hole. One is to activate sleeping nodes or place new nodes, and the other is to optimize the placement of the existing nodes. Due to the cost, many WSNs do not have sleeping nodes, and it is also difficult to put new nodes. Coverage holes can only be repaired by the dynamic node placement optimization. Therefore, the research on the dynamic node placement optimization algorithm is necessary.

The number of nodes in large-scale WSN is very large. This makes the node placement optimization a high-dimensional optimization problem with high requirements for the optimization algorithm. However, it takes a long time for most heuristic optimization algorithms to converge to the global optimal solution when solving high-dimensional optimization problems. Therefore, most of the global optimization algorithms cannot meet the time requirement for dynamic node placement optimization.

To solve this problem, a regional optimization dynamic algorithm for node placement is proposed. The proposed algorithm only optimizes the placement of the nodes in the selected region to reduce the dimension of the optimization problem. In addition, the unbalanced node residual energy and the energy consumption caused by node movement are rarely considered in existing research, which are under consideration in this paper.

The rest of the paper is organized as follows. Section 2 presents related work. An improved optimization model for regional optimization of node placement is proposed in Section 3. In Section 4, a regional optimization dynamic algorithm for node placement is proposed, including the re-optimized judgement conditions and the strategies for optimization region. Section 5 compares and analyzes the results of the simulation experiments. Finally, some conclusions are drawn in Section 6.

## 2. Related Work

Before node placement optimization, the location information and energy information of sensor nodes in the WSN should be collected. The energy information of sensor nodes will be transmitted to the sink node, while the location information of sensor nodes should be obtained by the node localization methods.

For outdoor node localization, the Global Positioning System (GPS) can help with the node localization. However, it is cost-expensive and not suitable for indoor environments because of its weak signal [8]. Wang et. al [9] used Received Signal Strength (RSS) measurements and proposed an approximate non-linear WLS estimator to solve the localization problem for both outdoor and indoor environments. To solve the indoor node localization problem, Garcia et al. [10] used Wireless Local Area Networks (WLANs) to deploy an indoor positioning system and proposed two approaches based on the Received Signal Strength Indicator (RSSI). Considering the generate reflections and refractions of wireless signals in indoor environments, Sendra et al. [11] developed a method for estimating indoor signal strength and proposed a wireless sensor placement system. Through these methods, the position information of sensor nodes in WSNs can be accurately obtained.

Once a node fault or death appears in the WSN, it can easily lead to a coverage hole and an energy hole. A lot of studies have been done on the problem of coverage hole repair.

Xu et al. [12] proposed an energy-efficient hole repair (EEHR) algorithm for WSN. EEHR wakes up the selected sleeping nodes by a triangle coverage repair procedure to repair the coverage hole. Chu et al. [13] proposed a tabu search (TS)-based network holes repair scheme. The proposed scheme is used to find the appropriate location to deploy new nodes to repair the coverage hole. However, these methods require the existence of sleeping nodes or new nodes in WSN. But in reality, many WSNs can only adjust the placement of existing nodes.

Zhuang et al. [14] used the invasive weed optimization algorithm (IWO) to strengthen the local search ability of differential evolution algorithm (DE), proposed a joint event coverage hole repair algorithm (JECHR) to optimize the node placement in WSN. JECHR can improve the coverage and reduce the moving distance effectively. Sharma et al. [15] proposed a hybrid differential evolution particle swarm optimization (DE-PSO) algorithm to reduce the residual node formation. DE-PSO has good global search ability and fast convergence which can be used to reduce the energy consumption and increase the lifetime of WSN. Wang et al. [16] proposed a non-dominated sorting multi-objective flower pollination algorithm (NSMOFPA). The non-linear convergence factor, the tent chaotic map, and a greedy crossover strategy are designed to improve the performance of the algorithm. NSMOFPA can be used to provide a better solution for node placement of WSN that has the objectives of coverage rate, node radiation overflow rate and energy consumption rate. However, these algorithms mentioned above are all global optimization algorithms for node placement. That is to say, the position of all the nodes in WSN will be moved within the whole monitoring area.

Hao et al. [17] proposed a three-dimensional coverage hole dynamic detection and repair algorithm. The proposed algorithm is adopted to move the redundant nodes adjacent to the coverage hole to redistribute the network. The moving direction and distance can be calculated according to the locations of redundant nodes and their neighbor nodes. However, this method can only move adjacent nodes to repair the coverage hole, and its performance is highly dependent on the density of nodes around the hole.

Khamlichi et al. [18] combined the gradient algorithm with a geographical-based approach to repair the identified coverage holes. This algorithm can be used to detect the redundant sensor nodes by calculating the Euclidean distance between the nodes, and then relocate them by using the gradient algorithm. Senouci et al. [19] proposed a distributed virtual forces-based local healing approach (HEAL). HEAL allows local healing where only the nodes located at an appropriate distance from the hole will be involved in the healing process. Khamlichi’s approach can reduce the number of moving nodes, and Senouci’s approach can reduce the scope of the optimization region. However, the unbalanced rest energy and energy consumption of moving nodes are not considered in both methods. Therefore, these approaches are not suitable for dynamic node placement optimization in WSN.

To overcome the limitations of the existing methods, this paper proposes a regional optimization dynamic algorithm for node placement in WSN.

## 3. Model for Regional Optimization of Node Placement

Traditional models for the node placement optimization problem in WSNs are mainly global optimization models. They take the coordinates or coordinates’ displacements of all the nodes in the network as the solution to the problem. In a global node placement optimization, the dimension of the problem is related to the total number of nodes in the network, and the search space of the solution is related to the size of the monitoring area. If the scale of a WSN is large, the node placement optimization problem will have a high dimension and a large search space. As a result, the complexity of the optimization algorithm is high so that the algorithm needs a longer time to converge.

Since the WSN has been running for a certain time when it needs to be re-optimized, the rest energy of each node in the network is different. It is not enough to optimize coverage only. This may result in the nodes with less energy moving farther, or moving to a position with heavy tasks and high energy consumption in the network topology. Therefore, it is necessary to take the coverage, energy consumption and energy balance, and the moving distance of nodes into consideration.

In this paper, an improved model for regional optimization of node placement is proposed based on the global node placement optimization model in [20]. The coordinates’ displacements of the nodes to be moved are taken as the solutions of the proposed model. The coverage of the whole network, the energy consumption and energy balance of the WSN (including energy consumption of network operation and nodes movement) are the objectives of the optimization problem. The equations of the optimization problem are shown in Equation (1).

(1) $$\begin{array}{cc} \; & \max\;f=[f_{1}\left(\Delta x_{r},\Delta y_{r}\right),f_{2}\left(\Delta x_{r},\Delta y_{r}\right)]\\ \; & s.t.\;\left\{\begin{array}{c} \Delta x_{r}\in \left(-a_{limit}x_{m},a_{limit}x_{m}\right)\\ \Delta y_{r}\in \left(-a_{limit}y_{m},a_{limit}y_{m}\right)\\ x_{r}\in \left(x_{dead}-r_{left},x_{dead}+r_{right}\right)\\ y_{r}\in \left(y_{dead}-r_{down},y_{dead}+r_{up}\right) \end{array}\right. \end{array}$$

where f represents the optimization objectives of the model, $f_{1}$ and $f_{2}$ respectively represent the two different optimization objectives of the optimization problem, $\Delta x_{r}=\left[\Delta x_{r}\left(1\right),\cdots ,\Delta x_{r}\left(i_{k}\right),\cdots ,\Delta x_{r}\left(n_{r}\right)\right]$ and $\Delta y_{r}=\left[\Delta y_{r}\left(1\right),\cdots ,\Delta y_{r}\left(i_{k}\right),\cdots ,\Delta y_{r}\left(n_{r}\right)\right]$ respectively represent the horizontal and vertical coordinates displacement row vectors of the nodes in the optimization region, $n_{r}$ is the number of the nodes in the optimization region, $1\leq i_{k}\leq n_{r}$, $x_{m}$ is the length of the monitoring area, $y_{m}$ is the width of the monitoring area, $\left(x_{dead},y_{dead}\right)$ is coordinate of the dead node, $a_{limit}$ is a coefficient that restricts the movement of nodes, $r_{left}$, $r_{right}$, $r_{down}$ and $r_{up}$ respectively represent the distance from the dead node to the left boundary, the right boundary, the lower boundary and the upper boundary of the optimized region.

$x_{r}=\left[x_{r}\left(1\right),\cdots ,x_{r}\left(i_{k}\right),\cdots ,x_{r}\left(n_{r}\right)\right]$ and $y_{r}=\left[y_{r}\left(1\right),\cdots ,y_{r}\left(i_{k}\right),\cdots ,y_{r}\left(n_{r}\right)\right]$ respectively represent the horizontal and vertical coordinate row vectors of the nodes in the optimization region. They can be calculated by Equation (2).

(2) $$\left\{\begin{array}{c} x_{r}\left(i_{k}\right)=\Delta x_{r}\left(i_{k}\right)+x_{0}\left(k_{r}\left(i_{k}\right)\right)\\ y_{r}\left(i_{k}\right)=\Delta y_{r}\left(i_{k}\right)+y_{0}\left(k_{r}\left(i_{k}\right)\right) \end{array}\right.$$

where $\left(x_{0}\left(k\right),y_{0}\left(k\right)\right)$ is the initial coordinate of the *k*th node, $k_{r}$ is a set of the nodes in the optimization region, $k_{r}\left(i_{k}\right)$ is the $i_{k}$th node in set $k_{r}$.

The coordinate of the *k*th node after optimization $\left(x_{k},y_{k}\right)$ can be calculated by Equations (3) and (4).

(3) $$x_{k}=\left\{\begin{array}{c} x_{r}\left(find\left(k_{r},k\right)\right)\;k\in k_{r}\\ x_{0}\left(k\right)\;\;\;k\notin k_{r} \end{array}\right.$$

(4) $$y_{k}=\left\{\begin{array}{c} y_{r}\left(find\left(k_{r},k\right)\right)\;k\in k_{r}\\ y_{0}\left(k\right)\;\;\;k\notin k_{r} \end{array}\right.$$

where $find(k_{r},k)$ represents the location of the *k*th node in $k_{r}$.

Coverage is the most important optimization target for node placement. It can measure the cover degree of sensor nodes to the monitoring area. Therefore, the coverage after optimization is the primary objective of the regional optimization problem.

To calculate the coverage of a WSN, the monitoring area can be discretized into pixels. If a pixel is covered, the square area represented by that pixel is considered to be covered. When there are enough pixels, the pixel matrix can be equated with the original monitoring area. It can be considered that the coverage calculated by the discretization employed for the pixels is accurate. The size of the pixel depends on the sensing range $r_{s}$ of the sensor nodes and the area $x_{m}\times y_{m}$ of the monitoring area. In this paper, a $x_{m}\times y_{m}$ monitoring area consists of a pixel matrix with $x_{m}$ rows and $y_{m}$ columns. The pixel point of row *i*, column *j* is the (i,j) pixel ($0<i\leq x_{m}$, $0<j\leq y_{m}$).

Boolean model (0-1 model) [21] is adopted in this paper. Obviously, the death nodes should be excluded when calculating the coverage. $k_{d}$ is used to put the serial numbers of death nodes. When the $k_{dead}$th node dies, this node should be added into $k_{d}$. The equations of the first objective are shown in Equations (5)–(8).

(5) $$f_{1}\left(\Delta x_{r},\Delta y_{r}\right)=Coverage=\frac{\sum P_{i,j}}{x_{m}\times y_{m}}$$

(6) $$d_{k}\left(i,j\right)=\sqrt{{\left(i-x_{k}\right)}^{2}+{\left(j-y_{k}\right)}^{2}}$$

(7) $$p_{i,j}\left(k\right)=\left\{\begin{array}{c} 1\;d_{k}\left(i,j\right)\leq r_{s}\\ 0\;else \end{array}\right.$$

(8) $$P_{i,j}=1-\prod_{k\notin k_{d}}\left(1-p_{i,j}\left(k\right)\right)$$

where $x_{m}$ and $y_{m}$ are the numbers of pixels in each row and column since the area of unit length unit width can be regarded as a pixel point, $p_{i,j}\left(k\right)$ represents whether the (i,j) pixel is covered by the *k*th node, $P_{i,j}$ represents whether the (i,j) pixel is covered by the WSN, $d_{k}\left(i,j\right)$ is the distance from the pixel point (i,j) to the *k*th node, $r_{s}$ is the sensing range of the sensor nodes, $k_{d}$ is a set of all death nodes.

After running for a period of time, the rest energy of the nodes in the WSN is unbalanced. If the nodes with low energy are ordered to move for a long distance, the rest energy of these nodes will be too low and die quickly. Moreover, the location of a node in a network topology can affect the speed of its energy consumption. The nodes with low energy are not suitable for some positions. Therefore, the energy consumption and energy balance in the WSN should be taken as one of the optimization objectives.

The energy consumption of each node includes the energy consumptions of movement and network operation. The energy consumption and energy balance of the network can be represented by the rest energy of the node with the least energy except all the death nodes. The rest energy of the node with least energy after the WSN continues running *r* rounds is calculated to predict the lifetime of the WSN. Since the WSN has been running for $r_{dead}$ rounds when a node dead, the continuous running round *r* should be related to $r_{dead}$. The equations of the second objective are shown in Equations (9)–(11).

(9) $$f_{2}\left(\Delta x_{r},\Delta y_{r}\right)=\min_{k\notin k_{d}}\left[E_{rest}\left(k,r\right)-E_{d}\cdot dis\left(k\right)\right]$$

(10) $$dis\left(k\right)=\left\{\begin{array}{ll} \sqrt{\Delta x_{r}{\left(find\left(k_{r},k\right)\right)}^{2}+\Delta y_{r}{\left(find\left(k_{r},k\right)\right)}^{2}} & k\in k_{r}\\ 0 & k\notin k_{r} \end{array}\right.$$

(11) $$r=\min(r_{max}-r_{dead},r_{min})$$

where $E_{rest}\left(k,r\right)$ is the rest energy of the *k*th node after *r* rounds, $E_{d}$ is an energy consumption coefficient of node movement, dis(k) is the moving distance of the *k*th node, $r_{min}$ is the minimum round required to predict the lifetime of the WSN, $r_{max}$ is the maximum round required to predict the lifetime of the WSN.

The values of $r_{min}$ and $r_{max}$ are related to the lifetime of the initial WSN. These two parameters are used to ensure that the WSN will not have many remaining nodes die after running *r* rounds.

## 4. Regional Optimization Dynamic Algorithm for Node Placement

The common dynamic node placement algorithm is to redistribute the location of all the nodes in the whole monitoring area. However, the death of one node only has a large impact on its surroundings. Moving all the nodes may cause unnecessary energy consumption. Therefore, when one node in WSN dies suddenly, the node placement optimization can be carried out on the nodes in a selected region. In this paper, a regional optimization dynamic algorithm for node placement is proposed. The proposed algorithm can be used to realize the regional node placement optimization while a WSN operating.

The key to the regional optimization dynamic algorithm is to obtain an appropriate node placement optimization region and use an optimization algorithm to redistribute the nodes in this region. It can optimize the performance of WSN by moving part of the nodes. Compared with the general node placement optimization algorithm, the regional optimization algorithm can greatly reduce the number of nodes that need to be moved, thus reducing the dimension of the problem, narrowing the search space of solution and reducing the complexity of the algorithm. Furthermore, the regional optimization algorithm only moves part of the nodes, which effectively reduces the energy consumption caused by node movement.

### 4.1. Re-Optimized Judgement Conditions for Node Placement

The sensor nodes may have faults in the process of operation, resulting in the inability to collect and transmit data. This kind of nodes can be regarded as dead nodes. A dead node may result in the loss of coverage or has little impact due to redundancy. Therefore, when a node dies, the information of WSN should be collected first to judge whether it is necessary to re-optimize the node placement.

**Figure 1:**
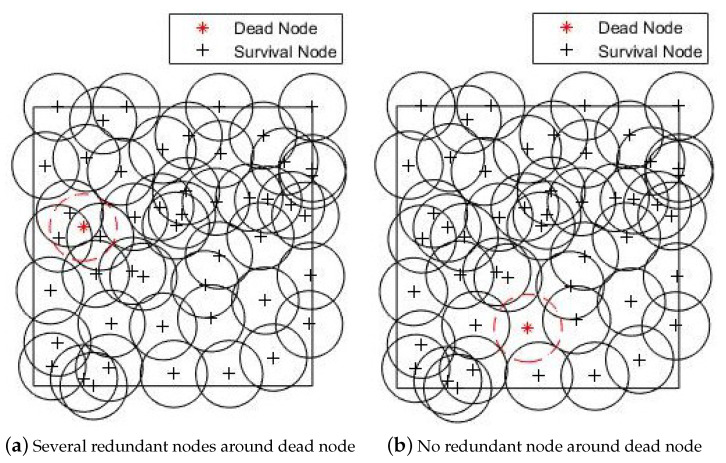
Figures with dead node at different positions.

The influence of node death is largely related to the node placement around the dead node. When a node dies, the identity number of the dead node $k_{dead}$ and its position coordinates $\left(x_{dead},y_{dead}\right)$ could be obtained. Figure 1a,b show two different dead nodes at different positions in the same initial node placement.

As shown in Figure 1a, if there are other nodes redundancy around the position of the dead node, the coverage of the entire WSN remains unchanged or only changes little. The node placement needs no optimization in this case. As shown in Figure 1b, if there are few nodes around the position of the dead node, the death of the node can lead to a coverage hole and even an energy hole. The node placement needs to be re-optimized in this case.

On the other hand, when the node placement is re-optimized, sensor nodes will consume energy due to movement. If the WSN has already run for a long time and the rest energy of each node is little, the energy of some nodes maybe not enough for moving. Re-optimization of node placement will result in more nodes deaths. Therefore, the remaining energy of all nodes $E_{\mathrm{re}}$ should be collected when one node dies. If there is another survival node with low remaining energy, it is not suitable to re-optimize the node placement.

For all these reasons, the re-optimized judgement conditions for node placement are shown in Equation (12).

(12) $$\left\{\begin{array}{c} \Delta Coverage>\varepsilon_{1}\\ \min_{k\notin k_{d}}E_{\mathrm{re}}>\varepsilon_{2}\cdot E_{o} \end{array}\right.$$

where ΔCoverage is the decreasing value of coverage due to the death of the $k_{dead}$th node, $\min_{k\notin k_{d}}E_{\mathrm{re}}$ is the minimum remaining energy of the current survival nodes (nodes except for set $k_{d}$), $E_{o}$ is the initial energy of all the nodes, $\varepsilon_{1}$ and $\varepsilon_{2}$ are constants of threshold coefficient.

The values of $\varepsilon_{1}$ and $\varepsilon_{2}$ are dependent on the application requirements of WSN. If full coverage is required, set $\varepsilon_{1}=0$; otherwise, the value of $\varepsilon_{1}$ should be related to the maximum coverage of one single sensor node, $0<\varepsilon_{1}\leq \frac{\pi \cdot r_{s}^{2}}{x_{m}\cdot y_{m}}$, where $\pi \cdot r_{s}^{2}$ represents the sensing area of one single sensor node and $x_{m}\cdot y_{m}$ represents the total area of the monitoring area. If WSN has a high coverage requirement, the value of $\varepsilon_{2}$ should be low; on the contrary, if WSN has a long lifetime requirement, the value of $\varepsilon_{2}$ should be high, $0\leq \varepsilon_{2}<1$. In this paper, we set $\varepsilon_{1}=0.1\cdot \frac{\pi \cdot r_{s}^{2}}{x_{m}\cdot y_{m}}$, $\varepsilon_{2}=0.1$.

### 4.2. The Strategies for Optimization Region

The regional optimization dynamic algorithm for node placement needs to obtain an appropriate optimization region and optimize the distribution of nodes in this region. Obviously, the optimization region is the key to the regional optimization dynamic algorithm. In this section, several strategies for optimization region are proposed.

#### 4.2.1. Surrounding Strategy for Optimization Region

The surrounding strategy for optimization region is a strategy to construct a region around the dead node as the optimization region. This strategy takes the position of the dead node as the center, $2r_{x}$ as the side length to construct a square area as the optimization region.

The value of $r_{x}$ is very important in the surrounding strategy for optimization region. If $r_{x}$ is too small, the area of the optimization region is also very small and there may have few nodes in the region. When the nodes in the optimization region are too sparse, the performance of WSN cannot be improved after the regional optimization for node placement. If $r_{x}$ is too big, the area of the optimization region is also very big. As a result, the effect of the surrounding strategy for optimization region is not obvious, and the regional node placement optimization is not much different from the global node placement optimization.

The optimization region should contain a sufficient number of nodes to prevent other coverage holes after the regional node placement optimization. Therefore, the value of $r_{x}$ can be determined according to the node density around the dead node. The sensing range $r_{s}$ can be used as the step length to gradually expand the range of the optimization region adaptively.

The main process of the surrounding strategy for optimization region is shown in Algorithm 1.
**Algorithm 1:** Surrounding Strategy for Optimization Region**In**:  Sensing range $r_{s}$, Length of the monitoring area $x_{m}$, Width of the monitoring area $y_{m}$, Location of all nodes (x,y), Dead node $k_{dead}$, Death nodes set $k_{d}$, Total number of nodes *n***Out**:Distances to regional boundary Region1, Set of nodes to be moved $k_{r1}$, Number of nodes to be moved $n_{r1}$
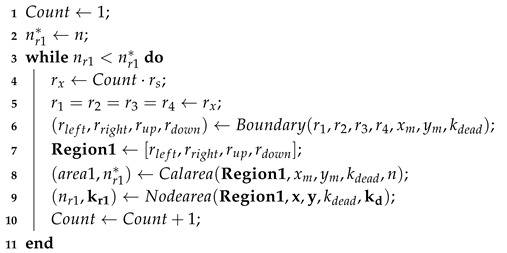


Some details in the pseudo-code in Algorithm 1 are explained below:

(1) Boundary
  Boundary can modify the boundary of the optimization region according to the position of the dead node, so that the optimization region will not exceed the monitoring area of WSN. The distances from the position of the dead node to the boundary of the optimization region can be calculated by Equation (13).
  (13) $$\left\{\begin{array}{c} r_{left}=\min\left(r_{1},x_{dead}\right)\\ r_{right}=\min\left(r_{2},x_{m}-x_{dead}\right)\\ r_{down}=\min\left(r_{3},y_{dead}\right)\\ r_{up}=\min\left(r_{4},y_{m}-y_{dead}\right) \end{array}\right.$$
  where $r_{1}$, $r_{2}$, $r_{3}$ and $r_{4}$ respectively represent the distances between the position of the dead node and the left boundary, right boundary, lower boundary and upper boundary of the optimization region. In the surrounding strategy for optimization region, $r_{1}=r_{2}=r_{3}=r_{4}=r_{x}$.
(2) Calarea
  Calarea is used to calculate the area of the optimization region area1 and the theoretical number of nodes $n_{r1}^{*}$ in this region. The equations are shown in Equations (14) and (15).
  (14) $$area1=\left(r_{left}+r_{right}\right)\cdot \left(r_{down}+r_{up}\right)$$
  (15) $$n_{r1}^{*}=n^{*}\cdot \frac{area1}{x_{m}\cdot y_{m}}$$
  where $n^{*}$ is the excepted node number of the whole monitoring area for an appropriate node density.
(3) Nodearea
  Nodearea is used to collect the information of the survival nodes in the optimization region, including the number of nodes $n_{r1}$ and the set of nodes $k_{r1}$.

#### 4.2.2. Redundant Strategy for Optimization Region

The redundant strategy for optimization region is a strategy to find the redundant nodes in WSN and construct the optimization region according to the position of the dead node and a redundant node. The first step of this strategy is to find a redundant node closest to the dead node. Then the positions of the dead node and the selected redundant node will be taken as two corners, and a rectangular region will be constructed as the optimization region for node placement.

The key to the redundant strategy is to select an appropriate redundant node. A method to calculate the overlapped number for the position of a node was proposed in [20]. The overlapped number Num(k) for the position $\left(x_{k},y_{k}\right)$ of the *k*th node can be calculated by Equation (16).

(16) $$Num\left(k\right)=\sum_{k_{i}\notin k_{d}}p_{x_{k},y_{k}}\left(k_{i}\right)$$

where $p_{i,j}\left(k\right)$ represents whether the pixel (i,j) is covered by the *k*th node.

Obviously, the larger Num(k) is, the denser the nodes around the *k*th node, and the more redundant the *k*th node is. When Num(k)≥3, the *k*th node can be considered to be a redundant node [20].

When the closest redundant node is selected, the optimization region for node placement will be constructed based on the position of the dead node $k_{dead}$ and the selected redundant node $k_{s}$. In order to prevent the dead node and the redundant node from being on the boundary, the length and width of the region should be appropriately increased. The distances between the position of the dead node and the boundaries of the optimization region can be calculated by Equation (17).

(17) $$\left\{\begin{array}{c} r_{1}=x_{dead}-\min\left(x_{k_{s}},x_{dead}\right)+2r_{s}\\ r_{2}=\max\left(x_{k_{s}},x_{dead}\right)-x_{dead}+2r_{s}\\ r_{3}=y_{dead}-\min\left(y_{k_{s}},y_{dead}\right)+2r_{s}\\ r_{4}=\max\left(y_{k_{s}},y_{dead}\right)-y_{dead}+2r_{s} \end{array}\right.$$

where $\left(x_{k_{s}},y_{k_{s}}\right)$ is the coordinate of the selected redundant node $k_{s}$.

In normal situations, the node density of any region can meet the requirements before a node dies. In this method, the constructed optimization region contains both the dead node and the selected redundant node. For a redundant node, even if it is removed, the node density in its region can still meet the requirements. That is to say, the redundant node can compensate for the loss of node density caused by the dead node. Therefore, after a node dies, the optimized region constructed by redundant strategy for optimization region can still meet the requirement of node density.

The main process of the redundant strategy for optimization region is shown in Algorithm 2.
**Algorithm 2:** Redundant Strategy for Optimization Region**In**:  Sensing range $r_{s}$, Length of the monitoring area $x_{m}$, Width of the monitoring area $y_{m}$, Location of all nodes (x,y), Dead node $k_{dead}$, Death nodes set $k_{d}$, Total number of nodes *n***Out**:Distances to regional boundary Region2, Set of nodes to be moved $k_{r2}$, Number of nodes to be moved $n_{r2}$_**1**_   
${\mathrm{Node}}_{r}\leftarrow FindRedundant\left(x,y,x_{m},y_{m}\right)$;
_**2**_   
$k_{s}\leftarrow Nearest\left({\mathrm{Node}}_{r},x,y,k_{dead}\right)$;
_**3**_   
$\left(r_{1},r_{2},r_{3},r_{4}\right)\leftarrow FindRegion\left(x,y,k_{dead},k_{s},r_{s}\right)$;
_**4**_   
$\left(r_{left},r_{right},r_{up},r_{down}\right)\leftarrow Boundary\left(r_{1},r_{2},r_{3},r_{4},x_{m},y_{m},k_{dead}\right)$;
_**5**_   
$\mathrm{Region}2\leftarrow [r_{left},r_{right},r_{up},r_{down}]$;
_**6**_   
$\left(n_{r2},k_{r2}\right)\leftarrow Nodearea\left(\mathrm{Region}2,x,y,k_{dead},k_{d}\right)$;


Some details in the pseudo-code in Algorithm 2 are explained below:

(1) FindRedundant
  FindRedundant can calculate the overlapped number Num(k) for each node in WSN by Equation (16), then put the nodes with Num(k)≥3 into redundant nodes set ${\mathrm{Node}}_{r}$.
(2) Nearest
  Nearest is used to calculate the distances between each node in ${\mathrm{Node}}_{r}$ and the dead node $k_{dead}$, then find out the closest redundant node $k_{s}$.
(3) FindRegion
  FindRegion can calculate the distances between the position of the dead node and the boundaries of the optimization region according to Equation (17).

#### 4.2.3. Mixed Strategy for Optimization Region

The surrounding strategy for optimization region can search the region around the dead node and gradually expand the optimization region until the node density meets the requirements. The redundant strategy for optimization region can find the position of the closest redundant node to the dead node and construct the optimization region to ensure the node density meets the requirements. Each of the two strategies has advantages and disadvantages. Therefore, the two strategies can be combined into a new mixed strategy for optimization region.

The main purpose of the regional optimization dynamic algorithm for node placement is to reduce the dimension and searching range of the algorithm. A good strategy for optimization region should be able to construct an optimization region with a small area, few nodes, and high node density. Both strategies can construct an optimization region that meets the requirements of node density. Therefore, the strategy with fewer nodes should be chosen because it can reduce the complexity of the algorithm more.

The time spent on the strategy for optimization region is very short and can be neglected. It is feasible to run both strategies first. Compare the numbers of nodes in the two optimization regions, $n_{r1}$ and $n_{r2}$. The optimization region with fewer nodes will be selected as the optimization region in the mixed strategy.

### 4.3. Process of MRDA

**Figure 2:**
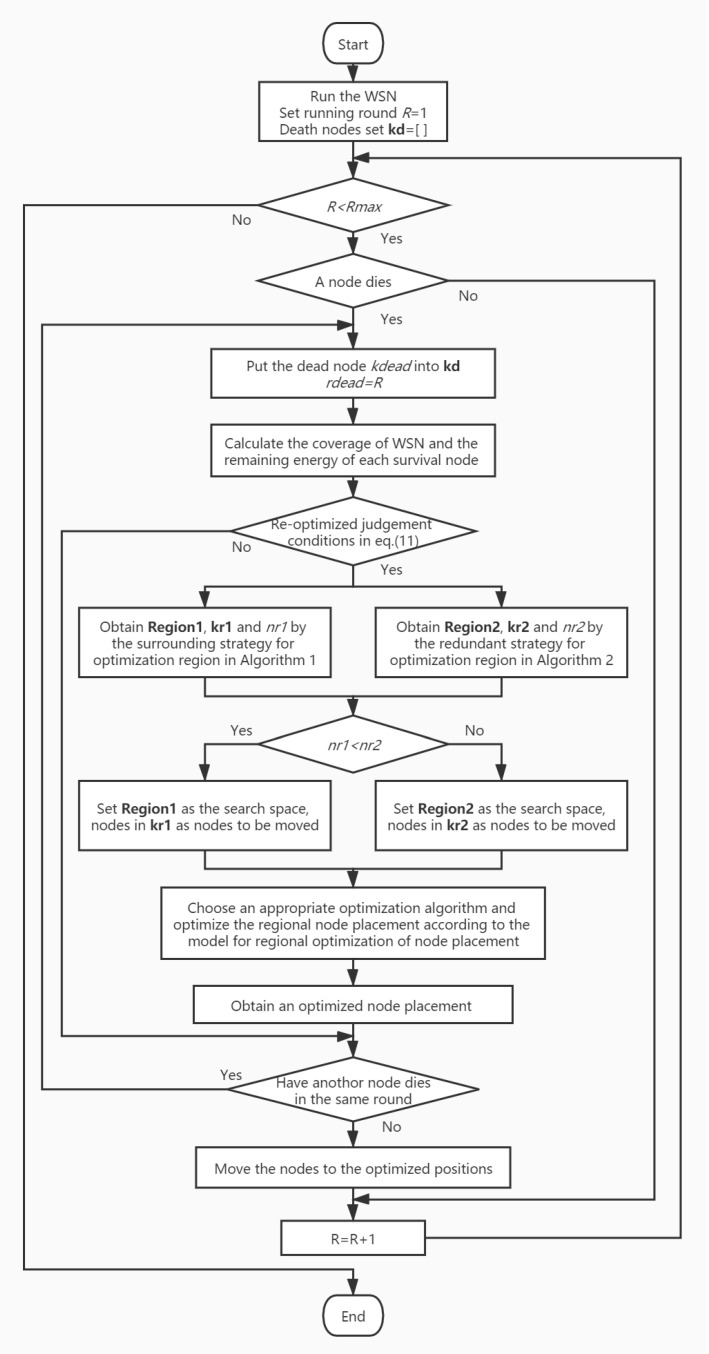
The flow chart of MRDA.

The regional optimization dynamic algorithm with the mixed strategy for optimization region is called Mixed Regional Dynamic Algorithm (MRDA). The flow chart of MRDA is shown in Figure 2.

MRDA will monitor whether a node dies during the operation of the WSN. If a node dies, MRDA will judge whether the re-optimization is needed. If the WSN in this round meets the re-optimized judgement conditions, MRDA can construct an optimization region using the mixed strategy for optimization region and optimize the regional node placement. If there are several nodes dead in the same round, MRDA will optimize them one by one. The previous optimization results will be regarded as the existing node placement. The judgement and regional optimization of the next death node will be based on it. The nodes will move to the final optimized positions after all the optimizations in this round are completed.

MRDA can be combined with any optimization algorithms. In this paper, the adaptive directed evolved NSGA2 (ADENSGA) [20] is chosen as the global node placement optimization algorithm. MRDA based on ADENSGA is called MR-ADENSGA.

## 5. Results and Discussion

### 5.1. Experimental Environment and Evaluation Indicators

The experimental environment is introduced in this section.

In this paper, the simulation of WSN and the test of algorithms are realized by MATLAB (Matlab R2017a). All the programs are run on a Windows 7 operating system (64 bit).

The proposed algorithm is implemented in a centralized architecture, and the base station is responsible for the execution of the algorithm and broadcasting the moving plan of all the sensor nodes.

Adaptive Directed Evolved Non-dominated Sorted Genetic Algorithm (ADENSGA) [20] is chosen as the basic node placement optimization algorithm in this paper. ADENSGA is an improved multi-objective optimization algorithm base on NSGA2 [22]. In ADENSGA, a directed evolved crossover operator is designed to determine the crossover probability of each node according to the node density around it. Also, the adaptive range limit is proposed to limit the movement range of a node after a generation of evolution. Since ADENSGA is proposed to optimize the node placement of WSNs, it is selected as the global node placement optimization algorithm in this paper. The related parameters of ADENSGA used in this paper is cited from [20].

The regional optimization dynamic algorithm with the surrounding strategy based on ADENSGA (SR-ADENSGA), the regional optimization dynamic algorithm with the redundant strategy based on ADENSGA (RR-ADENSGA), regional optimization dynamic algorithm with the mixed strategy based on ADENSGA (MR-ADENSGA), global node placement optimization algorithm ADENSGA with re-optimized judgement (J-ADENSGA), and the common global node placement optimization algorithm ADENSGA are tested in this section.

In this paper, Low Energy Adaptive Clustering Hierarchy (LEACH) is chosen as the routing protocol of WSNs. The energy consumption model and the related parameters of LEACH used in this paper is cited from [23].

The WSN for node placement optimization is composed of *n* sensor nodes distributed in the $x_{m}\times y_{m}$ monitoring area and a sink node outside the area. The node $k_{dead}$ suddenly dies in round $r_{dead}$. $r_{max}\approx 0.5r_{firstdead}^{*}$, $r_{min}\approx 0.25r_{max}$, $\varepsilon_{1}=0.1\cdot \frac{\pi \cdot r_{s}^{2}}{x_{m}\cdot y_{m}}$, $\varepsilon_{2}=0.1$ in this paper, where $r_{firstdead}^{*}$ is the round the first node dies when WSN is running normally.

Other parameters used in the experiment are shown in Table 1.

In this paper, the results of the non-dominated solutions are analyzed and compared. In ADENSGA, the population is sorted according to the level of non-domination. Domination means that all the objectives of one solution are superior to the other. A solution can be regarded as a non-dominated solution if it is not dominated by any other solutions. It is also one of the first dominance level solutions [22].

Besides the optimization objectives of the regional optimization for node placement, Coverage and RestEnergy, three additional evaluation indicators are used in this paper. The comprehensive score Score [20], the total moving distance of nodes *D* [20], and the increasing coverage–distance rate RD [24] can be calculated by Equations (18)–(20).

(18) $$Score=f_{1}\cdot f_{2}$$

(19) $$D=\sum_{k=1}^{n}dis\left(k\right)$$

(20) $$RD=\frac{\Delta Area}{D}$$

where $f_{1}$, $f_{2}$ are the optimization objectives of the problem shown in Equations (5) and (9), dis(k) is the moving distance of the *k*th node, *n* is the number of sensor nodes in the monitoring area, ΔArea is the increasing cover area of WSN.

The larger Score is, the better the optimization results of the algorithm are. The smaller *D* is, the less energy the node movement consumes, and the better the optimization result of the algorithm will be. The larger RD is, the greater the coverage growth caused by the movement of per unit distance, the more effective the node moving in the algorithm.

### 5.2. Experiments on Different Random Node Death

In this section, experiments are carried out on WSN with different random node death.

Set $x_{m}=y_{m}=100$, n=50, $r_{max}=400$, $r_{min}=100$. The $k_{dead}$th node dies in round $r_{dead}$, with the death nodes set $k_{d}$. Randomly set 5 different initial node placement, and randomly set 2 groups values of $k_{dead}$, $r_{dead}$ and $k_{d}$ for each of them. Each group of $k_{dead}$, $r_{dead}$ and $k_{d}$ represents a different situation. In each situation, the evaluation indicators without any optimization algorithms and with optimization algorithms are shown in Table 2. The results of MR-ADENSGA will be compared with ADENSGA, SR-ADENSGA and RR-ADENSGA. The average results and its standard deviations of all the non-dominated solutions in 5 runs are shown in Table 2, where the optimal results are highlighted in bold.

Table 2 shows that within 100 generations, SR-ADENSGA, RR-ADENSGA and MR-ADENSGA are all able to achieve better results than ADENSGA on Coverage, RestEnergy and Score. In addition, compared with ADENSGA, SR-ADENSGA, RR-ADENSGA and MR-ADENSGA can effectively reduce the dimension of the algorithms and the total moving distance of nodes *D*, and their increasing coverage–distance rate RD is also larger. This is due to the high dimension of ADENSGA and the randomness of predicting the value of RestEnergy. Therefore, ADENSGA cannot converge to the global optimal solution within limited generations.

When the node placement needs no optimization, the running time of SR-ADENSGA, RR-ADENSGA and MR-ADENSGA is very short and can be ignored. When the node placement needs to be re-optimized, the running time of ADENSGA, SR-ADENSGA, RR-ADENSGA and MR-ADENSGA is similar for the same generations. This is because that the running time of the algorithms mainly influenced by the predicting round *r* of WSN in $f_{2}$. Therefore, the algorithm with shorter convergence generation takes less time.

ADENSGA has no re-optimized judgement conditions. When the dead node is in a redundant position (Situation 4 and Situation 10), in order to improve the coverage of very few, ADENSGA must move a large distance, resulting in large energy consumption. When the remaining energy of the nodes in round $r_{dead}$ is very low (Situation 6), the remaining energy of the other nodes is very low, which is easy to cause new coverage holes.

Among the three regional optimization dynamic algorithms, SR-ADENSGA and RR-ADENSGA have unstable performance under different situations, while MR-ADENSGA can generally obtain the best results.

To further analyze the results in Table 2, several typical situations are selected. The optimization regions of each algorithm and the curves of the evaluation indicators changing with generations are presented below.

**Figure 3:**
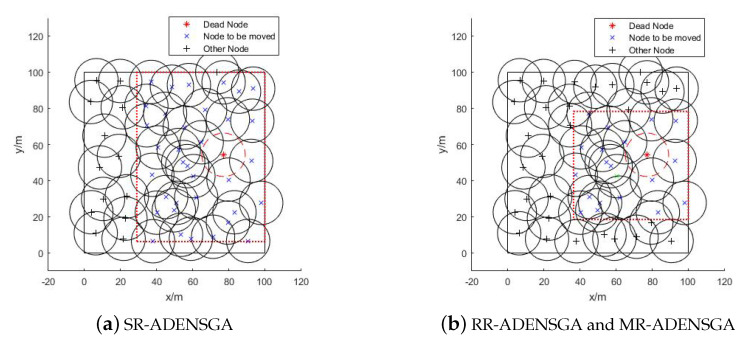
Optimization Region and Position of Nodes to be Moved in Situation 1.

The optimization region and the position of the nodes to be moved of different algorithms in Situation 1 are shown in Figure 3. The dotted box represents the optimization region, and ’×’ represents the node to be moved. Figure 3a shows the optimization region of SR-ADENSGA, Figure 3b shows the optimization region of RR-ADENSGA and MR-ADENSGA. The optimization region of ADENSGA is the entire monitoring area, and all the remaining nodes will be moved.

Figure 3 shows that in Situation 1, the node density around the position of the dead node is low. In this case, the RR-ADENSGA has a smaller optimization region and fewer nodes than SR-ADENSGA. The strategy for optimization region of MR-ADENSGA is the same as RR-ADENSGA. In addition, RR-ADENSGA and MR-ADENSGA have a higher node density in the optimization region. Therefore, the performance of RR-ADENSGA and MR-ADENSGA is better than SR-ADENSGA.

**Figure 4:**
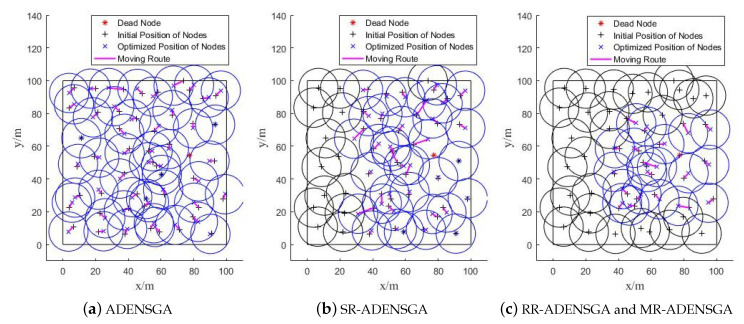
One of the Optimization Solutions to Different Algorithms in Situation 1.

Figure 4 shows one of the optimization solutions to different algorithms in Situation 1. Among them, Figure 4a shows the solution to ADENSGA, Figure 4b shows the solution to SR-ADENSGA, Figure 4c shows the solution to RR-ADENSGA and MR-ADENSGA. The initial positions, optimized positions and the motion paths of nodes are shown in Figure 4.

**Figure 5:**
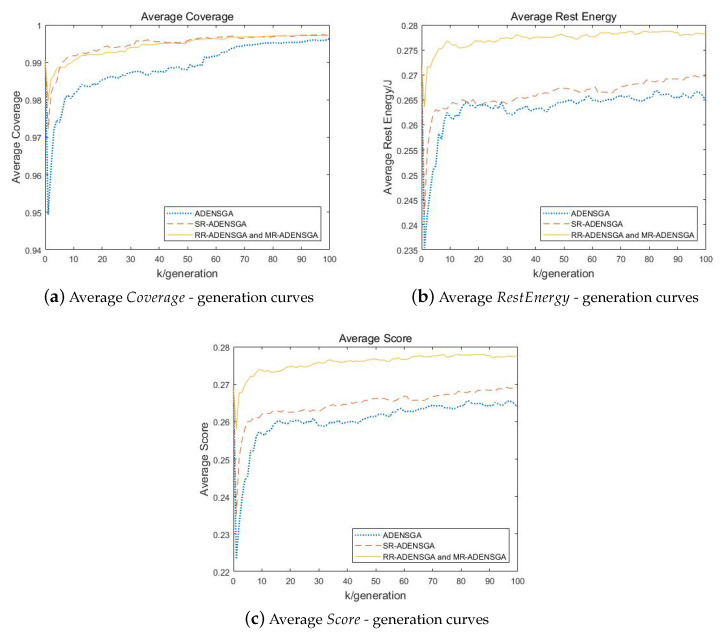
Comparison of Optimization Results of Different Algorithms Changing With Generation in Situation 1.

Figure 5 shows the results of ADENSGA, SR-ADENSGA, RR-ADENSGA and MR-ADENSGA in each generation in Situation 1. Among them, Figure 5a shows the average Coverage—generation curves, Figure 5b shows the average RestEnergy—generation curves, Figure 5c shows the average Score—generation curves. The curves respectively represent the average of the mean value of the first dominance level’s Coverage, RestEnergy and Score changing with generation in 5 runs.

Figure 5 shows that the optimization performance and convergence generation of RR-ADENSGA and MR-ADENSGA are all far better than ADENSGA and SR-ADENSGA. This is because the optimization region of SR-ADENSGA is very large, resulting in poor effect of the regional optimization algorithm. RR-ADENSGA and MR-ADENSGA converge around the 60th generation, while ADENSGA and SR-ADENSGA cannot converge within 100 generations.

**Figure 6:**
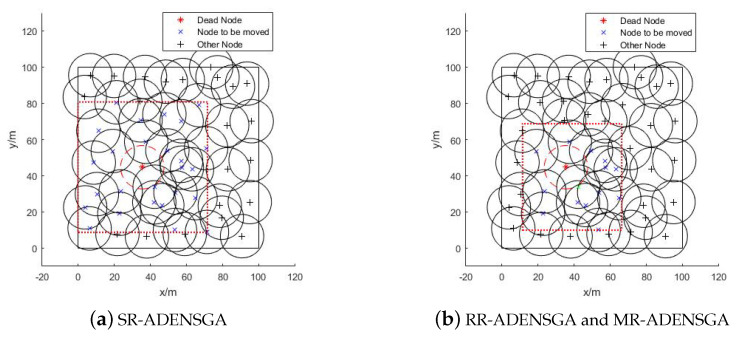
Optimization Region and Position of Nodes to be Moved in Situation 2.

The optimization region and the position of the nodes to be moved of different algorithms in Situation 2 are shown in Figure 6. Figure 6a shows the optimization region of SR-ADENSGA, Figure 6b shows the optimization region of RR-ADENSGA and MR-ADENSGA.

Figure 6 shows that in Situation 3, the node density around the position of the dead node is uneven. In this case, the RR-ADENSGA has a smaller optimization region and fewer nodes than SR-ADENSGA. The strategy for optimization region of MR-ADENSGA is the same as RR-ADENSGA. There is little difference in node density between the two optimization regions. Therefore, the performance of RR-ADENSGA and MR-ADENSGA is slightly better than SR-ADENSGA.

**Figure 7:**
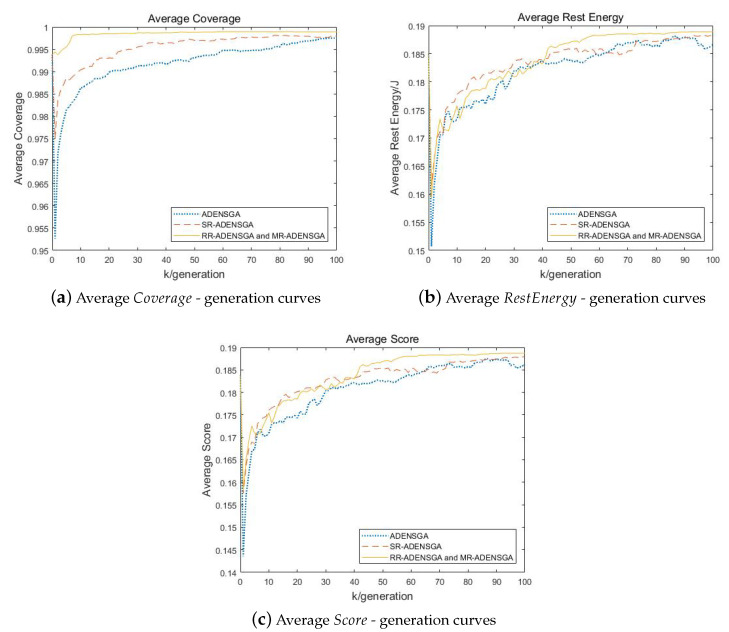
Comparison of Optimization Results of Different Algorithms Changing With Generation in Situation 2.

Figure 7 shows the results of ADENSGA, SR-ADENSGA, RR-ADENSGA and MR-ADENSGA in each generation in Situation 2. It shows that the optimization performance and convergence generation of SR-ADENSGA, RR-ADENSGA and MR-ADENSGA are all better than ADENSGA. However, due to the uneven node placement around the dead node, RR-ADENSGA and MR-ADENSGA can search much faster than SR-ADENSGA and ADENSGA. RR-ADENSGA and MR-ADENSGA converge around the 60th generation, while SR-ADENSGA and ADENSGA cannot converge within 100 generations.

**Figure 8:**
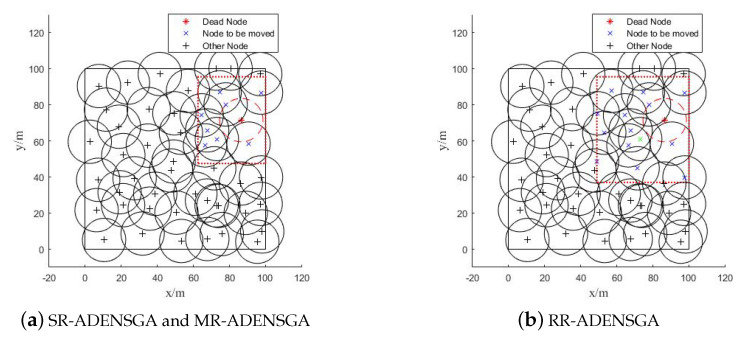
Optimization Region and Position of Nodes to be Moved in Situation 9.

The optimization region and the position of the nodes to be moved of different algorithms in Situation 9 are shown in Figure 8. Figure 8a shows the optimization region of SR-ADENSGA and MR-ADENSGA, Figure 8b shows the optimization region of RR-ADENSGA.

Figure 8 shows that in Situation 9, the node density around the position of the dead node is moderate. In this case, the SR-ADENSGA has a smaller optimization region and fewer nodes than RR-ADENSGA. The strategy for optimization region of MR-ADENSGA is the same as SR-ADENSGA. Although RR-ADENSGA has a bigger optimization region and more nodes, it has a higher node density in the optimization region. Therefore, the performance of RR-ADENSGA is similar to SR-ADENSGA and MR-ADENSGA.

**Figure 9:**
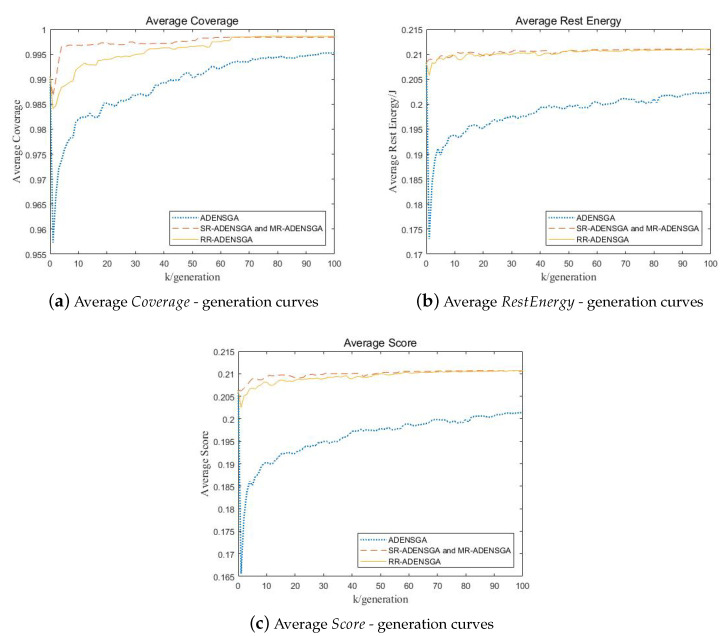
Comparison of Optimization Results of Different Algorithms Changing With Generation in Situation 9.

Figure 9 shows the results of ADENSGA, SR-ADENSGA, RR-ADENSGA and MR-ADENSGA in each generation in Situation 9. It shows that the optimization performance and convergence generation of SR-ADENSGA, RR-ADENSGA and MR-ADENSGA are all far better than ADENSGA. SR-ADENSGA and MR-ADENSGA converge around the 50th generation, RR-ADENSGA converges around the 70th generation, while ADENSGA cannot converge within 100 generations.

The average lifetimes of the WSNs optimized by ADENSGA, SR-ADENSGA, RR-ADENSGA and MR-ADENSGA are also compared in this section. The situations are the same as those in Table 2. The round of next node dead, the round of half node dead and the round of all node dead are collected to represent the lifetime of WSNs. The average results of all the non-dominated solutions in 5 runs in these 10 situations are shown in Table 3, where the optimal results are highlighted in bold.

Table 3 shows that WSNs optimized by SR-ADENSGA, RR-ADENSGA and MR-ADENSGA within 100 generations have longer lifetimes than those optimized by ADENSGA. In addition, WSNs optimized by MR-ADENSGA have the longest lifetimes. This is consistent with the results of RestEnergy. Therefore, the proposed algorithm can effectively prolong the lifetime of WSNs.

In conclusion, the regional optimization dynamic algorithm can optimize the node placement of WSN faster and better within limited generations. It can reduce the dimension of the problem and narrow the search space so that the proposed algorithm can converge faster. Both SR-ADENSGA and RR-ADENSGA can construct an appropriate node placement optimization region. They perform unstably in different situations. MR-ADENSGA combines the advantages of both algorithms and can construct a better optimization region. Therefore, the optimization effect, convergence speed and adaptability of MR-ADENSGA is the best.

### 5.3. Experiments on Different Scales of WSNs

In this section, experiments are carried out on WSNs with different scales.

Due to differences of coverage and remaining energy between different WSNs, coverage and rest energy cannot be chosen as the evaluation indicators directly. In this experiment, the increasing coverage and increasing rest energy are chosen as the evaluation indicators. The increasing coverage means the increasing value of coverage after optimization. The increasing rest energy means the increasing value of rest energy after optimization.

Set $x_{m1}=y_{m1}=100$ when $n_{1}=50$, $x_{m2}=y_{m2}=140$ when $n_{2}=100$, $x_{m3}=y_{m3}=170$ when $n_{3}=150$, $x_{m4}=y_{m4}=200$ when $n_{1}=200$. Set $r_{max}\approx 0.5r_{firstdead}^{*}$, $r_{min}\approx 0.25r_{max}$.

**Figure 10:**
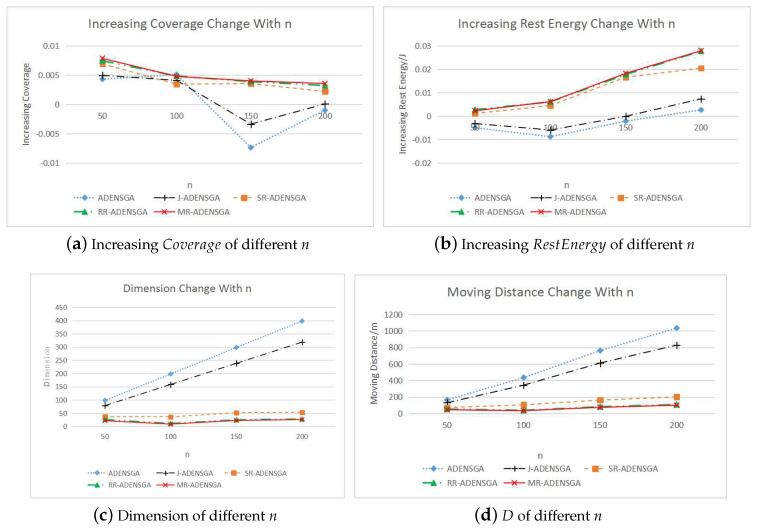
Comparison of Optimization Results for WSNs with Different Scales.

When $n\in [n_{1},n_{2},n_{3},n_{4}]$, the WSNs with *n* sensor nodes will have a node death after a certain round. The results of MR-ADENSGA will be compared with ADENSGA, J-ADENSGA, SR-ADENSGA and RR-ADENSGA. For WSNs of each scale, the optimization experiment will be carried out in 5 different situations (including 1 situation of no re-optimization). The increasing values of Coverage, the increasing values of RestEnergy, the dimensions of the algorithms and the total moving distance *D* will be compared. The average results of all the non-dominated solutions are shown in Figure 10.

Figure 10a,b show that for WSNs with different scales, MR-ADENSGA can achieve the best optimization performance within limited generations. Compared with ADENSGA and J-ADENSGA, SR-ADENSGA and RR-ADENSGA have better and more stable optimization performance. Therefore, the strategies for optimization region is effective. Compared with ADENSGA, J-ADENSGA has a better optimization performance. That is to say, the re-optimized judgement conditions for node placement are effective. The proposed regional optimization dynamic algorithm for node placement can quickly compensate for the defects caused by the dead node by adjusting the distribution of part of the nodes.

Figure 10c shows that the dimensions of solutions of SR-ADENSGA, RR-ADENSGA and MR-ADENSGA are much smaller than ADENSGA and J-ADENSGA, while the dimensions of J-ADENSGA is slightly smaller than ADENSGA. The more nodes in WSN, the greater the dimension difference. This proves that the proposed algorithm can effectively reduce the dimension, thus reducing the complexity of the algorithm and shortening the optimization time.

Figure 10d shows that the moving distances of SR-ADENSGA, RR-ADENSGA and MR-ADENSGA are much smaller than ADENSGA and J-ADENSGA, while the moving distance of J-ADENSGA is slightly smaller than ADENSGA. The more nodes in WSN, the greater the moving distance difference. This proves that the proposed algorithm can effectively reduce the energy consumption caused by node movement, thus prolonging the lifetime of WSN.

In addition, Figure 10 shows that MR-ADENSGA combines the advantages of SR-ADENSGA and RR-ADENSGA, and performs best in all evaluation indicators.

In conclusion, for the node placement problem of large-scale WSNs, the proposed regional optimization dynamic algorithm has obvious advantages. The re-optimized judgement conditions for node placement can help avoid some unnecessary optimizations, and the strategies for optimization region can help reduce the dimension and obtain better optimization results within limited generations. Among the three strategies proposed in Section 4.2, the mixed strategy for optimization region has the best optimization performance and adaptability.

### 5.4. Experiments on Different Node Densities of WSNs

Node density has a great impact on the node placement and node redundancy of WSNs. In this section, experiments are carried out on WSNs with different node densities.

Node density is the number of nodes per unit area, as shown in Equation (21).

(21) $$nodedensity=\frac{n}{x_{m}\cdot y_{m}}$$

Set $x_{m}=y_{m}=100$, nodedensity∈[0.004,0.005,0.006,0.007]. Therefore, $n_{5}=40$, $n_{6}=50$, $n_{7}=60$, $n_{8}=70$. Set $r_{max}\approx 0.5r_{firstdead}^{*}$, $r_{min}\approx 0.25r_{max}$.

**Figure 11:**
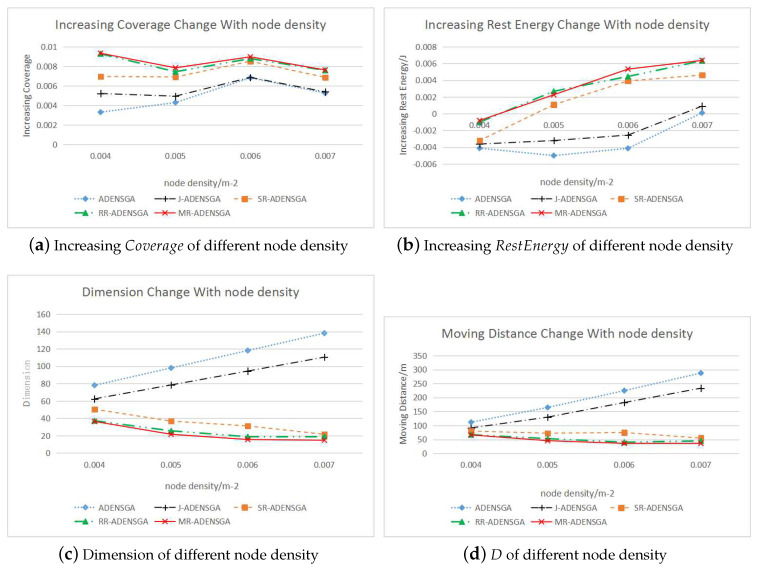
Comparison of Optimization Results for WSNs with Different Node Densities.

When $n\in [n_{5},n_{6},n_{7},n_{8}]$, the WSNs with *n* sensor nodes will have a node death after a certain round. The results of MR-ADENSGA will be compared with ADENSGA, J-ADENSGA, SR-ADENSGA and RR-ADENSGA. For WSNs of each node density, the optimization experiment will be carried out in 5 different situations (including 1 situation of no re-optimization). The increasing values of Coverage, the increasing values of RestEnergy, the dimensions of the algorithms and the total moving distance *D* will be compared. The average results of all the non-dominated solutions are shown in Figure 11.

Figure 11a,b show that for WSNs with different node densities, MR-ADENSGA can achieve the best optimization performance within limited generations. Compared with ADENSGA, J-ADENSGA and SR-ADENSGA, RR-ADENSGA has a better and more stable optimization performance. When the node density is high, SR-ADENSGA performs better than ADENSGA and J-ADENSGA. When the node density is low, SR-ADENSGA performs similarly to ADENSGA and J-ADENSGA. Due to the low node density, SR-ADENSGA will degenerate to J-ADENSGA in most situations. Therefore, the proposed mixed strategy for optimization region is effective for any node densities. Compared with ADENSGA, J-ADENSGA has a better optimization performance. That is to say, the re-optimized judgement conditions for node placement are effective.

Figure 11c shows that the dimensions of solutions of SR-ADENSGA, RR-ADENSGA and MR-ADENSGA are much smaller than ADENSGA and J-ADENSGA, while the dimensions of J-ADENSGA is slightly smaller than ADENSGA. The higher the node density is, the greater the dimension difference. This proves that the proposed algorithm can effectively reduce the dimension, thus achieving better results within limited generations.

Figure 11d shows that the moving distances of SR-ADENSGA, RR-ADENSGA and MR-ADENSGA are much smaller than ADENSGA and J-ADENSGA, while the moving distance of J-ADENSGA is slightly smaller than ADENSGA. The higher the node density is, the greater the moving distance difference. This proves that the proposed algorithm can effectively reduce the energy consumption caused by node movement, thus prolonging the lifetime of WSN.

In addition, Figure 11 shows that MR-ADENSGA combines the advantages of SR-ADENSGA and RR-ADENSGA, and performs best in all evaluation indicators.

In conclusion, for the node placement problem of high node density, the proposed regional optimization dynamic algorithm has obvious advantages. The surrounding strategy for optimization region performs poorly in WSNs with low node density. Among the three strategies proposed in Section 4.2, the mixed strategy for optimization region has the best optimization performance and adaptability.

## 6. Conclusions

To solve the coverage hole and energy hole caused by node fault or node death during the WSN operation, a regional optimization dynamic algorithm is proposed in this paper.

In this paper, a model for regional optimization of node placement and a regional optimization dynamic algorithm for node placement are proposed. The proposed model takes into account the factors of coverage, unbalanced node residual energy, and energy consumption caused by node movement. The proposed algorithm can judge whether the node placement needs to be re-optimized when a node suddenly dies, and then obtain an appropriate optimization region with nodes to be optimized. Surrounding strategy and redundant strategy for optimization region are proposed, then a mixed strategy for optimization region is proposed. The regional optimization dynamic algorithm with the mixed strategy for optimization region (MRDA) can be combined with any optimization algorithms. Therefore, MRDA has good universality.

In this paper, coverage and energy consumption (including energy consumption of network operation and nodes movement) are the two objectives of the node placement optimization problem. The ADENSGA-based MRDA (MR-ADENSGA) is compared with other algorithms. Experiments show that the proposed algorithm can greatly reduce the dimension and the moving distance, with a significant improvement in the search performance and convergence speed in WSNs with any node density that meets the requirements. The proposed algorithm performs better in large-scale WSNs.

## Figures and Tables

**Table 1:** Parameter Settings.

Coordinates of the sink node, $(x_{sink},y_{sink})/m$	$\left(0.5x_{m},1.75y_{m}\right)$
Node movement range coefficient, $a_{limit}$	0.15
Sensing range of the node, $r_{s}/m$	12
Initial energy of nodes, $E_{o}/J$	0.5
Energy consumption coefficient of node movement, $E_{d}/\left(J/m\right)$	5 × 10^−3^
Excepted node number for an appropriate node density, n*	50
Size of the population, $P_{size}$	20
Maximum generation of the algorithm, $k_{max}$	100

**Table 2:** Optimization Results for Different $k_{dead}$ and $r_{dead}$.

Situations	Algorithms	Dimension of Solutions	Average Coverage	Average RestEnergy /J	Average Score	Average D/m	Average RD/m	Average Running Time /s
Situation 1 Node Placement 1 $k_{dead}=8$ $r_{dead}=250$ $k_{d}=\left[8\right]$	No optimization	-	0.9907	0.2718	0.2693	-	-	-
	ADENSGA	98	0.9935 ± 0.0020	0.2631 ± 0.0071	0.2614 ± 0.0066	164.5 ± 2.5	0.1702	123.3
	SR-ADENSGA	70	0.9963 ± 0.0037	0.2734 ± 0.0014	0.2724 ± 0.0012	127.2 ± 5.0	0.4403	124.5
	RR-ADENSGA	**40**	**0.9991 ± 0.0002**	**0.2786 ± 0.0008**	**0.2783 ± 0.0007**	**82.0 ± 3.8**	**1.0244**	**121.9**
	MR-ADENSGA	**40**	**0.9991 ± 0.0002**	**0.2786 ± 0.0008**	**0.2783 ± 0.0007**	**82.0 ± 3.8**	**1.0244**	**121.9**
Situation 2 Node Placement 1 $k_{dead}=36$ $r_{dead}=380$ $k_{d}=\left[8,36\right]$	No optimization	-	0.9938	0.1850	0.1839	-	-	-
	ADENSGA	96	0.9975 ± 0.0006	0.1868 ± 0.0042	0.1863 ± 0.0041	146.8 ± 3.2	0.2520	193.7
	SR-ADENSGA	52	0.9980 ± 0.0003	0.1884 ± 0.0008	0.1880 ± 0.0007	96.3 ± 3.1	0.4361	194.5
	RR-ADENSGA	**28**	**0.9989 ± 0.0002**	**0.1889 ± 0.0004**	**0.1887 ± 0.0004**	**48.9 ± 1.7**	**1.0440**	**192.0**
	MR-ADENSGA	**28**	**0.9989 ± 0.0002**	**0.1889 ± 0.0004**	**0.1887 ± 0.0004**	**48.9 ± 1.7**	**1.0440**	**192.0**
Situation 3 Node Placement 2 $k_{dead}=5$ $r_{dead}=180$ $k_{d}=\left[5\right]$	No optimization	-	0.9806	0.2973	0.2915	-	-	-
	ADENSGA	98	0.9880 ± 0.0094	0.3026 ± 0.0109	0.2989 ± 0.0098	165.4 ± 12.7	0.4474	157.3
	SR-ADENSGA	**22**	**0.9924 ± 0.0000**	**0.3123 ± 0.0001**	**0.3099 ± 0.0001**	**45.9 ± 0.6**	**2.5708**	**154.4**
	RR-ADENSGA	40	0.9917 ± 0.0005	0.3122 ± 0.0005	0.3096 ± 0.0003	83.0 ± 5.4	1.3373	156.3
	MR-ADENSGA	**22**	**0.9924 ± 0.0000**	**0.3123 ± 0.0001**	**0.3099 ± 0.0001**	**45.9 ± 0.6**	**2.5708**	**154.4**
Situation 4 Node Placement 2 $k_{dead}=28$ $r_{dead}=90$ $k_{d}=\left[28\right]$	No optimization	-	0.9918	0.3441	0.3413	-	-	-
	ADENSGA	98	**0.9935 ± 0.0035**	0.3366 ± 0.0070	0.3344 ± 0.0059	176.9 ± 9.9	**0.0961**	178.3
	SR-ADENSGA	**0**	0.9918	**0.3441**	**0.3413**	**0**	0	**0.0059**
	RR-ADENSGA	**0**	0.9918	**0.3441**	**0.3413**	**0**	0	0.0090
	MR-ADENSGA	**0**	0.9918	**0.3441**	**0.3413**	**0**	0	0.0150
Situation 5 Node Placement 3 $k_{dead}=41$ $r_{dead}=330$ $k_{d}=\left[41\right]$	No optimization	-	0.9772	0.2186	0.2136	-	-	-
	ADENSGA	98	0.9910 ± 0.0005	0.2031 ± 0.0092	0.2017 ± 0.0091	173.0 ± 6.1	0.7977	**129.9**
	SR-ADENSGA	94	0.9881 ± 0.0027	0.2149 ± 0.0036	0.2125 ± 0.0031	181.5 ± 9.4	0.6006	133.5
	RR-ADENSGA	**24**	**0.9915 ± 0.0018**	**0.2220 ± 0.0007**	**0.2198 ± 0.0005**	**47.9 ± 1.9**	**2.9854**	132.4
	MR-ADENSGA	**24**	**0.9915 ± 0.0018**	**0.2220 ± 0.0007**	**0.2198 ± 0.0005**	**47.9 ± 1.9**	**2.9854**	132.4
Situation 6 Node Placement 3 $k_{dead}=24$ $r_{dead}=600$ $k_{d}=\left[41,24\right]$	No optimization	-	0.9835	0.0415	0.0408	-	-	-
	ADENSGA	96	**0.9924 ± 0.0034**	0.0316 ± 0.0052	0.0313 ± 0.0041	157.2 ± 8.7	**0.5661**	197.3
	SR-ADENSGA	**0**	0.9835	**0.0415**	**0.0408**	**0**	0	0.0129
	RR-ADENSGA	**0**	0.9835	**0.0415**	**0.0408**	**0**	0	**0.0079**
	MR-ADENSGA	**0**	0.9835	**0.0415**	**0.0408**	**0**	0	0.0209
Situation 7 Node Placement 4 $k_{dead}=19$ $r_{dead}=220$ $k_{d}=\left[19\right]$	No optimization	-	0.9907	0.2754	0.2728	-	-	-
	ADENSGA	98	0.9932 ± 0.0056	0.2693 ± 0.0080	0.2675 ± 0.0067	175.6 ± 7.1	0.1425	144.7
	SR-ADENSGA	**16**	**0.9993 ± 0.0001**	**0.2802 ± 0.0002**	**0.2800 ± 0.0002**	**34.0 ± 2.0**	**2.5294**	**142.2**
	RR-ADENSGA	20	0.9992 ± 0.0003	**0.2802 ± 0.0002**	**0.2800 ± 0.0002**	47.6 ± 4.4	1.7857	**142.2**
	MR-ADENSGA	**16**	**0.9993 ± 0.0001**	**0.2802 ± 0.0002**	**0.2800 ± 0.0002**	**34.0 ± 2.0**	**2.5294**	**142.2**
Situation 8 Node Placement 4 $k_{dead}=32$ $r_{dead}=290$ $k_{d}=\left[19,32\right]$	No optimization	-	0.9859	0.2238	0.2206	-	-	-
	ADENSGA	96	0.9910 ± 0.0045	0.2291 ± 0.0046	0.2270 ± 0.0037	162.8 ± 6.7	0.3132	200.7
	SR-ADENSGA	78	0.9898 ± 0.0053	0.2249 ± 0.0089	0.2226 ± 0.0079	147.3 ± 6.6	0.2648	199.9
	RR-ADENSGA	**20**	**0.9978 ± 0.0006**	**0.2342 ± 0.0002**	**0.2337 ± 0.0000**	**38.0 ± 2.6**	**3.1316**	**198.3**
	MR-ADENSGA	**20**	**0.9978 ± 0.0006**	**0.2342 ± 0.0002**	**0.2337 ± 0.0000**	**38.0 ± 2.6**	**3.1316**	**198.3**
Situation 9 Node Placement 5 $k_{dead}=22$ $r_{dead}=350$ $k_{d}=\left[22\right]$	No optimization	-	0.9905	0.2083	0.2063	-	-	-
	ADENSGA	98	0.9955 ± 0.0053	0.1992 ± 0.0068	0.1983 ± 0.0063	172.1 ± 10.5	0.2901	**129.8**
	SR-ADENSGA	**16**	**0.9985 ± 0.0005**	**0.2113 ± 0.0003**	**0.2109 ± 0.0004**	**24.9 ± 0.2**	**3.2129**	133.3
	RR-ADENSGA	28	0.9981 ± 0.0042	0.2109 ± 0.0003	0.2105 ± 0.0008	57.6 ± 1.7	1.3194	132.0
	MR-ADENSGA	**16**	**0.9985 ± 0.0005**	**0.2113 ± 0.0003**	**0.2109 ± 0.0004**	**24.9 ± 0.2**	**3.2129**	133.3
Situation 10 Node Placement 5 $k_{dead}=49$ $r_{dead}=120$ $k_{d}=\left[49\right]$	No optimization	-	0.9971	0.3353	0.3343	-	-	-
	ADENSGA	98	**0.9989 ± 0.0007**	0.3338 ± 0.0022	0.3334 ± 0.0021	162.9 ± 3.9	**0.1105**	167.1
	SR-ADENSGA	**0**	0.9971	**0.3353**	**0.3343**	**0**	0	**0.0044**
	RR-ADENSGA	**0**	0.9971	**0.3353**	**0.3343**	**0**	0	0.0070
	MR-ADENSGA	**0**	0.9971	**0.3353**	**0.3343**	**0**	0	0.0115

**Table 3:** Average Lifetimes for Different Algorithms.

	ADENSGA	SR-ADENSGA	RR-ADENSGA	MR-ADENSGA
Average Round of Next Node Dead	671.2	681.3	683.9	**684.3**
Average Round of Half Node Dead	852.0	861.3	864.4	**868.5**
Average Round of All Node Dead	1334.6	1383.5	1367.1	**1383.6**

## Abbreviations

The following abbreviations are used in this manuscript:

WSN	Wireless sensor network
LEACH	Low Energy Adaptive Clustering Hierarchy
NSGA2	Fast Non-dominated Sorted Genetic Algorithm
